# Supplementary material for: Long-term neuron tracking reveals balance of stability and plasticity in functional properties
Source: PLoS One. 2026 Jun 29;21(6):e0321830. doi: 10.1371/journal.pone.0321830 (PMC13313350; doi:10.1371/journal.pone.0321830)
Supplement: Attachment — Submitted filename: NT - R1 - point-by-point - final.docx [file pone.0321830.s003.docx]

**Long-term neuron tracking reveals balance of stability and plasticity in functional properties**
Point-by-point response

We sincerely thank the editors and reviewers for their detailed feedback and suggestions on our manuscript. We are honored that Reviewer #2 believes that “the manuscript is largely straightforward and the methods reported here could be useful to researchers using classic MEA probes” and Reviewer #3 believes that “The manuscript is nicely and clearly written and presents the information in a clear and concise manner. The data analysis is sound, and the authors utilized stringent criteria to select those neurons that would be considered to be the same across different recording sessions, showing that their intrinsic functional properties were preserved”. We have made substantial revisions to the manuscript that incorporate all the information and additional material required by the reviewers. The revision has greatly improved the quality of the paper and clarified our core concepts. Below we have addressed each of the reviewers’ comments individually and highlighted where corresponding changes have been made in the manuscript.

**Reviewer #2:**
In this manuscript the authors report a method to track single-unit waveforms recorded from classic MEAs across sessions and days. They start with neurons that were ‘online’ sorted by their recording system within a session and use a waveform similarity metric to track these sorted neurons across days/sessions. The underlying algorithm is relatively simple, it’s a pairwise comparison of mean neural waveforms. Specifically, it computes the euclidean distance and the pearson correlation, normalizes it, and takes the mean of these two metrics. The similarity is compared to a null-distribution and if it is significant the neural waveforms from different days are ‘merged’. Using this method, they can track ~40% of their total sorted neurons across multiple days. The median is close to ~20 days, but a subset were tracked over ~100 days. For these tracked neurons, they analyze the mean firing-rates, inter-spike-intervals, tuning direction and field coupling and state that these properties are mostly stable (although see comments). The major strengths of this paper are the long-term neural recordings, and the manuscript is largely straightforward and the methods reported here could be useful to researchers using classic MEA probes. The main weakness of the paper is that the lack of comparisons to other methods make it hard to determine how well the method works, and the biological insight gained from these analyses is very limited, and although the results here are clear - they could use some more analyses, nuance and controls.

We thank the reviewer for appreciating this manuscript and giving strong feedback to strengthen the paper. We have revised the manuscript accordingly.

**Major comments:**
1.How good is this method compared to other approaches? The lack of a ‘ground truth’ makes it difficult to evaluate how much better this method is or how effective it is. A meaningful comparison to other methods - i.e. ones that use other features or different metrics would make the effectiveness of this system more obvious and a clear validation step would be useful.

We thank the reviewer for the excellent suggestion to benchmark our approach against existing methods. To address this, we directly compared our algorithm with UnitMatch (*1*), a well-regarded method designed specifically for high-density probes such as Neuropixels. Importantly, UnitMatch provides a publicly available dataset (*2*), allowing a transparent and reproducible head-to-head evaluation.

Although both approaches leverage waveform similarity to identify neurons across sessions, they were developed for fundamentally different recording regimes. UnitMatch is optimized for high-density arrays, where individual neurons are simultaneously detected across multiple nearby channels. This enables the use of spatial metrics such as decay similarity, centroid position, and trajectory that are inherently unavailable in the single- or low-channel recordings for which our algorithm is designed. Thus, the comparison is not meant to determine superiority, but rather to clarify use cases and highlight complementary strengths.

In the UnitMatch dataset, five rodents were implanted with chronic Neuropixels probes, each with two sessions collected one day apart. Besides the original UnitMatch procedure, we applied our algorithm to the same data. Across subjects, our method recovered many within-day matches, suggesting that it generalizes well even to data outside its intended domain. As expected for an approach that does not use spatial information, our sensitivity (0.144 ± 0.042) was lower than UnitMatch’s (0.454 ± 0.009). However, our specificity remained perfect (1.0000 ± 0.0000), matching UnitMatch’s performance (0.9992 ± 0.0005).

We view this result positively and believe it reflects deliberate design choices rather than a limitation. First, our method prioritizes high specificity, which is essential for longitudinal tracking: false positives have far more damaging consequences than missed matches, particularly in clinical or multi-session cognitive studies. Second, the benchmark dataset contains only two sessions per subject and therefore does not capture the temporal variability across months-long datasets that our algorithm was built for. Third, because UnitMatch’s decisions rely heavily on spatial features unavailable in our data domain, the sensitivity difference primarily reflects sensor constraints rather than algorithmic weakness. Finally, UnitMatch provides a useful benchmark but not a definitive ground truth. Some matches labeled as correct by UnitMatch may be overly liberal, meaning our algorithm’s “misses” may in fact represent correct rejections.

Overall, this comparison demonstrates that our method maintains excellent specificity and produces reasonable matches even in a dataset optimized for a different type of electrode array. This supports the robustness of our approach and clarifies the scenarios in which it provides advantages: long-term, multi-session studies with conventional MEAs where spatial features are not available. We have included this in the Results section.

2. The system seems to heavily rely on online-sorted/pre-sorted neurons, which can have their own error rates etc - however, more modern tools combine sorting and tracking these steps into a single computation, which allows them to capture drift over the session - could the authors comment on why they chose their approach?

We thank the reviewer for this critical feedback. Indeed, there are plenty of tools that combine sorting and tracking. Indeed, several modern spike sorting frameworks do combine sorting and drift tracking into a single computational procedure. Examples include Kilosort (*3*), SpyKING CIRCUS 2.0 (*4*), MountainSort 4 (*5*), and Herdingspikes2 (*6*), all of which incorporate template evolution or explicit spatial drift correction during the sorting process. While these tools jointly perform spike sorting and drift correction, they are designed primarily for within-session drift and rely heavily on high-density spatial information, such as that provided by Neuropixels probes. These approaches solve a different problem from the one addressed in our work.

First, our goal is to identify neuron identity across sessions and across days, not just track sub-second or minute-scale drift within a single session. Most of these sorters, such as Kilosort or FAST (*7*), assume temporal continuity: templates evolve gradually during a single uninterrupted recording. However, they do not infer correspondence across discontinuous sessions, where electrodes may have been reconnected, tissue may have shifted substantially, and waveform appearance may change abruptly.

Second, drift-correcting sorters depend on spatial features that are unavailable in conventional MEAs, such as classic microwire arrays, Utah arrays, or tetrode-based systems. These settings were operated by many laboratories, including ours. Tools like Kilosort or SpyKING CIRCUS leverage information such as depth-based template drift, multi-channel spatial footprint, and consistent relative channel amplitudes. In low-density systems, each neuron appears on only one channel, making these methods unsuitable for cross-day identity tracking in such datasets.

Third, even modern joint-sorting approaches do not guarantee cross-day “identity stability.” Their merging decisions are typically optimized for accurate sorting within a recording session. Without explicit cross-session modeling, the same biological neuron recorded across different days will usually be labeled as different units, despite good sorting quality.

Finally, our algorithm is designed for the challenges inherent to multi-session, multi-day chronic recordings, such as those encountered in human intracranial recordings or long-term animal experiments. Our focus is on developing a consistent and interpretable metric space in which units from different days can be compared even when the waveform shapes differ due to biological or recording-related changes. This differs fundamentally from real-time drift correction and complements, rather than replaces, existing sort-and-track methods.

Taken together, while joint sorting-tracking tools have advanced within-session accuracy, they do not address the key question targeted by our work: how to robustly identify neurons across discontinuous recording sessions using conventional electrode arrays. Our approach directly tackles this gap.

3. For the more biologically relevant results reported here, some more comparative analysis is warranted. The role of the task in the results reported here is largely ignored - There are two kinds of neurons in these recordings, neurons that are used for the kalman filter and presumably other unrelated neurons - which wouldn’t have a pd. A comparative analysis for these two kinds of neurons would have been a useful discovery - are the neurons that are task related the more stable ones? And the ones which are not task related are the ones that go silent or have larger changes? Are there any consistent trends about which neurons shift and which dont? Is it related to task performance, does stability depend on how informative that neural response was for the kalman filter?

We thank the reviewer for this thoughtful suggestion. We agree that understanding how task involvement relates to tuning stability is biologically meaningful. However, in our dataset, most tracked units were incorporated into the Kalman decoder at least some of the time (e.g., 72/114 for Monkey A; 293/353 for Monkey B). As a result, a strict binary comparison between “task-related” and “task-unrelated” neurons is not feasible because very few neurons were never used.

To address the reviewer’s concern, we instead quantified task involvement continuously, using the fraction of sessions in which each neuron contributed to the Kalman filter. This metric captures the degree to which a neuron participated in task control across days. We then compared this measure with each neuron’s PD span.

Across animals, we found weak to moderate relationships (Spearman ρ = 0.32 in Monkey A; ρ = 0.59 in Monkey B), indicating that neurons used more frequently in the decoder tend to have slightly smaller PD spans, but that task usage explains only a modest portion of the variance. This result suggests that tuning stability is not exclusively a property of the “direct” population.

Importantly, extensive BMI literature shows that neurons not explicitly used in the decoder (“indirect neurons”) nonetheless develop task-related modulation and exhibit significant tuning changes during learning. This phenomenon has been demonstrated in classic work showing network-wide plasticity (*8*, *9*), population-dynamics studies showing adaptation across the entire manifold (*10*), and long-term learning studies documenting reorganization in both direct and indirect units (*11*). These findings corroborate our observation that neurons outside the decoder can still exhibit meaningful directional tuning and stability.

4. I am having a hard time understanding how one can claim stability when the PD for a neuron changes over 200 degrees over time. This shows stability in the neural recording, not in the neural responses. For determining PDs did the authors use a threshold for finding neurons that had a good fit to the cosine model? The shifting PDs could also be a sign of assigning PDs to neurons that aren’t actually directionally tuned.

We thank the reviewer for raising this point and appreciate the opportunity to clarify our definition of PD stability. We are not claiming that a neuron with a 200° change in preferred direction (PD) is “stable” in an absolute sense. Instead, stability in our manuscript is defined relative to a null distribution generated from random PD trajectories over the same duration. The PD span therefore reflects whether a neuron’s directional preference changes less than expected by chance, not whether the absolute magnitude of its change is small. This distinction may not have been sufficiently clear in the original text, and we have now revised the manuscript to emphasize this point.

**Regarding the concern about directional tuning, we fully agree that PD estimates are only meaningful when the cosine model provides a reliable fit. To quantify tuning quality, we computed bootstrap confidence intervals (CIs) for each neuron’s PD. Poorly tuned neurons exhibit large PD CIs due to high uncertainty in the cosine fit. Across the population, we found that PD estimates were generally reliable: the median 95% CI widths were 58.43° for Monkey A and 33.36° for Monkey B, indicating good fits overall.**

**To ensure that downstream analyses were based only on well-tuned units, we excluded neurons with PD CI widths greater than 120°. This threshold removed 224 units in Monkey A (34.91%) and 375 units in Monkey B (22.81%). We regenerated the results in Figure 4 using only these well-tuned neurons (**Reviewer Response Figure 1**) as well as Figure 7 (**Reviewer Response Figure 2**). We found that all major conclusions remained unchanged. Applying this criterion therefore strengthens our analysis by ensuring that neurons with unreliable directional tuning do not influence PD stability estimates.**

**Reviewer Response Figure 1. Revised Figure 4 in the manuscript.** (A) The changes in PDs between neighboring sessions in tracked neurons (Monkey A: N=316, p=0.329; Monkey B: N=1045, p=0.75. One-sample t-test). (B) The numbers and percentages of tracked neurons that had stable and unstable PDs, as characterized by the PD span. (C) The PDs and their corresponding mean waveforms of an example tracked neuron.

**Reviewer Response Figure 2. Revised Figure 7 in the manuscript.** The Venn diagrams showed the numbers of tracked neurons in each factorial combination of the stability of ISI, PD, and SFC. Almost all of tracked neurons were stable in at least one functional property (107 out of 116 tracked neurons in Monkey A and 323 out of 354 tracked neurons in Monkey B), whereas more than 30% of tracked neurons have stable ISI, PD, and SFC (25% for Monkey A and 32% for Monkey B).

5. Was there any behavioral consequence for having stable neurons? Right now this is largely a phenomenological study, it could help if the authors had a biological hypothesis this allowed one to test.

We thank the reviewer for raising this important conceptual point. Our biological hypothesis in this work is that individual neurons exhibit quantifiable stability (or instability) in their preferred direction over long timescales, and that such stability can be rigorously measured using our tracking framework. Thus, the primary goal of the study is to characterize the structure of PD stability across months of intracortical recordings rather than to manipulate stability to test its behavioral consequences.

Our current dataset does not permit a causal analysis of how stable versus unstable neurons influence behavior because (a) the decoder used almost all available units (see Reviewer #2, Major comment 3), leaving no experimental manipulation of which neurons contributed to control, and (b) neural stability can only be assessed retrospectively after many sessions have accrued. Thus, behavioral differences conditioned on neuron stability cannot be assessed within this dataset.

However, we agree with the reviewer that the framework opens the door to mechanistic tests in future experiments. One concrete extension would be to explicitly compare behavioral performance when decoding is driven by “stable” versus “plastic” neurons, as identified using long-term tracking. In a BMI paradigm, one could construct decoders using only neurons with historically stable PDs or only neurons with historically unstable PDs. This would allow a causal test of whether stability facilitates faster learning, smoother control, or reduced recalibration requirements. Such an experiment would directly probe the functional consequences of neural stability and represents a promising direction enabled by the present methodology.

We have added this discussion to the revised manuscript.

For BMI applications, tracking neurons can enhance decoder stability and calibration. A persistent challenge in BMI design is neural signal drift or loss, which necessitates frequent recalibration of decoders (58) that takes time every session (59) and may impede recruitment of neural plasticity (3). By identifying stable neurons over time, our approach could facilitate the development of adaptive BMIs that compensate for gradual neural changes, improving long-term performance and reducing patient effort in maintaining control. Another extension of this study would be to explicitly compare behavioral performance when decoding is driven by “stable” versus “plastic” neurons, as identified using long-term tracking. In a BMI paradigm, one could construct decoders using only neurons with historically stable PDs or only neurons with historically unstable PDs. This would allow a causal test of whether stability facilitates faster learning, smoother control, or reduced recalibration requirements. Finally, our findings could guide investigations into intrinsic neural variability, such as changes in ISI distributions that may reflect ion channel regulation or synaptic plasticity. Understanding the biological mechanisms underlying neural stability and plasticity could lead to new insights into the fundamental principles governing motor control and learning. Future work should aim to refine tracking methodologies, extend recording durations, and explore the relationship between neural stability and motor learning to further advance our understanding of cortical dynamics over time.

**Minor comments**
1. It’s not clear to me why you would use the mean of the pearson correlation and the euclidean distance for similarity scores, instead of treating them as a 2D vector - as the authors themselves point out, these capture two different aspects of similarity.

We thank the reviewer for this thoughtful point. Pearson correlation and Euclidean distance indeed capture complementary aspects of waveform similarity. Our aim in this work was to use a similarity score that is easy to interpret, stable across sessions, and computationally lightweight, given that our primary contribution is the cross-session tracking framework rather than the definition of a new waveform metric. Averaging the two measures provides a single scalar that preserves contributions from both shape-based (correlation) and amplitude-based (distance) similarity, while avoiding an additional optimization or classifier layer that would add complexity.

**Treating the two metrics as a 2D vector is an interesting direction. In principle, one could model this space using clustering or a supervised similarity learner, but this would require choosing a weight, a classifier, or distance metric in the 2D space, which implicitly collapses the dimensions in another way. Because our empirical tests indicated that the simple averaged metric already produced stable, high-quality matches, we elected to use this straightforward formulation in the interest of interpretability and reproducibility.**

2. The reasoning for comparison of mean firing rates across sessions was not clear to me, wouldn’t comparing only the first and last sessions, bias your estimates toward stability since many neurons are recorded for <20 days? Was there a measurement of session by session mean firing rate changes, maybe a regression analysis here would clarify how the number of sessions alters firing rates?

We thank the reviewer for this insightful comment. To address the concern that comparing only the first and last sessions might bias the analysis toward neurons recorded for fewer days, we examined whether the magnitude of firing-rate change depended on the duration between the first and last recorded session for each neuron. Specifically, we regressed the ratio of last-session to first-session firing rate against the number of days spanned by the recording. We found only weak relationships between recording duration and firing-rate change (Monkey A: Pearson’s r = –0.21; Monkey B: r = 0.15, Reviewer Response Figure 3).

We also grouped neurons by the number of days between their first and last sessions and compared the mean firing-rate change across these bins. Consistent with the correlation analysis, we did not observe systematic differences across bins, and the mean firing-rate changes did not differ significantly between subjects (p > 0.05). These results indicate that the first–versus–last comparison does not preferentially bias the analysis toward neurons recorded for short durations.

**Reviewer Response Figure 3.** The relationship between changes in firing rates and the duration of the neuron.

3. In general, readability would have been improved if the results section used more declarative titles that described the result instead of a generic title. For e.g. instead of titles like “Neuron tracking”, something more along the lines of “Neurons were tracked for xx days” etc.

We thank the reviewer for this suggestion. We have changed the title accordingly.

Neurons were tracked for more than 100 days

4. The copy I received for review had the figure legends inserted at odd locations into the main text, please check that the submission is appropriately formatted before sending it out to review.

We thank the reviewer for noting this issue and apologize for any inconvenience caused by the formatting. The misplaced figure legends likely resulted from the automated PDF compilation used during the submission process. In the revised version, we placed all the figure captions at the end of the document.

5. Specific text suggestions: “Neural stability is essential for executing learned … “ While I understand the general sentiment behind this statement, this dichotomy is not true. Degeneracy in neural circuits enables skilled behavior even though neural firing patterns are not identical. E.g. EMG activity (and therefore motor neuron activity) can vary substantially, but the hand kinematics can be identical. For cortical studies, the Null-Space shown by kaufman and colleagues is another example of neural acitivity in individual neurons not being deterministic for behavior. There are more examples for other flexible behaviors such as the STG patterns of neural activity shown by Prinz and Marder and in other invertebrates. It would be prudent to dial back these dichotomies. For stable/unstable representations since the authors cited the Liberti et al 2016 paper, they should also cite the Katlowitz et al 2018 neuron paper which challenges the observations in the Liberti paper.

We thank the reviewer for this thoughtful perspective and for highlighting the importance of degeneracy and flexibility in neural circuits. We agree that stable behavior does not require perfectly stable single-neuron activity, and that phenomena such as EMG variability, null-space activity in motor cortex, and circuit-level degeneracy in systems such as the crustacean stomatogastric ganglion (STG) illustrate this well. In the revised manuscript, we have softened the dichotomous language in the Abstract and expanded the Discussion to situate our work within this broader context. We also now cite Katlowitz et al. (2018) alongside Liberti et al. (2016) to better represent the full range of findings on stable vs. variable neural representations.

Neural circuits can support stable behaviors even as their underlying activity patterns evolve, reflecting a balance between robustness and flexibility in response to learning and changing conditions (1–4).

**Reviewer #3:**
In the present study, the authors implemented a novel methodology to track neural activity across recording sessions spread over many days (3 months), with the larger goal of assessing the long term stability of neural activity associated with the control of a robotic arm using a Brain-Machine Interface (BMI) which translated neural population spiking activity into cursor velocity, enabling macaques to perform a "center-out" task. Using their tracking approach, based in waveform similarity comparison across sessions, the authors were able to successfully track individual neurons across days and to assess the stability of their functional properties (direction tuning, Inter-Spike Interval distribution, and Spike-Field Coherence), and show that individual neurons exhibit a high degree of stability, while some plasticity is present, and hypothesize that the balance between stability and flexibility allow motor learning. The manuscript is nicely and clearly written, and presents the information in a clear and concise manner. The data analysis is sound, and the authors utilized stringent criteria to select those neurons that would be considered to be the same across different recording sessions, showing that their intrinsic functional properties were preserved. The main weakness of the manuscript is the lack of some sort of "ground truth" test. I believe it would be relatively easy to generate synthetic data that could be analyzed using the proposed tracking method. This would allow to also determine what kind of variations in the detected spikes may lead the tracking algorithm to fail. Another option would be to apply the tracking method to subsets of data taking randomly a fraction of the trials on each session, and assessing the consistency of the results.

We thank the reviewer for their overall enthusiasm for our manuscript. We have addressed the comments below and revised the manuscript accordingly.

We especially appreciate the reviewer pointing out that “the main weakness of the manuscript is the lack of some sort of ground truth test”. We agree that evaluating a tracking method in the absence of *true* ground truth is challenging, and we appreciate the suggestion to use synthetic data or subsampled sessions. Here, we incorporated a complementary validation analysis that addresses this concern while remaining aligned with the constraints of our dataset and recording hardware.

Instead of generating synthetic spikes, which inevitably reflect assumptions about noise structure, drift trajectories, and waveform variability that may not generalize, we elected to benchmark our method using a real, publicly available dataset with an established reference framework. Specifically, we performed a head-to-head comparison against UnitMatch (*1*) using the chronic Neuropixels recordings released by (*2*).

This dataset provides a practical proxy for “ground truth” by splitting each recording session into two halves, allowing within-day matches to serve as validated examples of correct identity assignments. This approach avoids imposing artificial generative models while providing an objective evaluation of tracking accuracy.

Our algorithm showed strong specificity and recovered many of the validated within-day matches, even though the dataset was recorded with high-density probes and includes spatial features unavailable in our own recordings. Importantly, we emphasize that our method is designed for conventional low-density MEAs, whereas UnitMatch relies heavily on spatial metrics that cannot be computed in our domain. Because of these fundamental differences, comparative analysis highlights complementary strengths rather than relative superiority. We have added this benchmarking analysis to the revised manuscript. For more details, please refer to our response to Reviewer #2, Major comment 1.

1. Please specify what kind of tungsten electrode arrays were used (i.e. manufacturer) and their characteristics (tip length, spacing, impedance range, etc.)

We thank the reviewer for clarifying the specifications of the electrode arrays. Below is the manufacture as well as their characteristics. We also included this information in the manuscript.

Each monkey was unilaterally implanted with tungsten microelectrode arrays (MEAs; Innovative Neurophyiology, Durham, NC; 35 um diameter, 6.5 mm length, 500 um pitch) targeting the primary motor cortex (M1) and premotor cortex (PMd). Monkey A was implanted with a 64-channel MEA, while Monkey B received a 128-channel MEA. The implantation sites were determined using stereotaxic coordinates, pre-operative magnetic resonance images, and cortical landmarks.

2. How stable were the units sorted during a given recording session. It would be useful for the reader to have an idea of the waveform stability. The authors mention that only units with high SNR were used for the analysis, it would be nice to know how stable the SNR was during a session, in addition to across sessions.

We appreciate this feedback from the reviewer. In the beginning of a session, we utilized the Ripple software to identify neurons through “hoops”, where the users define a fixed range that sets the permitted variability of spikes. Multiple hoops were used for each neuron to define more strictly regarding the waveforms of a neuron. Any spikes outside of the defined range were excluded, thus ensuring within-session stability.

3. It would be useful to indicate in Figure 1 that the log transformed Euclidean distance was used in the similarity matrix, to avoid confusion in the readers that may be thinking of correlation as "similarity" vs distance as "dissimilarity".

We thank the reviewer for this comment. We have revised the figure caption to more explicitly mention that log-transformed Euclidean distances and Fisher-transformed Pearson correlations were averaged as similarity.

The total similarity matrix (shape of 11 by 11; only upper triangular matrices was shown) was the average of two rescaled similarity metrics, including Fisher-transformed Pearson correlation and log-transformed Euclidean distance.

4.Line 440. "Monkey B exhibited significant changes in mean firing rates, whereas Monkey A did not." The distributions in Figure 2C look very similar. In fact, the distribution of relative changes in mean FR for monkey B peaks at zero, while the distributor monkey A peaks below 0. Therefore, this conclusion is hard to accept, at least without a statistic. Statistical test results are given for peak and through amplitude, but not for mean FR.

We appreciate the sharp observation from the reviewer. The statistical results for the mean firing rates were missing in the original manuscript. We have included that in the manuscript.

(C) The relative changes in peak amplitudes, trough amplitudes, and mean firing rates (Monkey A: peak amplitudes, p<0.05, Cohen’s D=0.215; trough amplitudes, p>0.05; firing rate, p>0.05. Monkey B: peak and trough amplitudes, p>0.05; firing rate, p<0.001, Cohen’s D=0.281).

5.Lines 481-484. "An example tracked neuron (Fig 4C) exhibited a PD around 37.6°, gradually shifting clockwise to 246.8° over 91 days, yet its PD span (150.8°) remained well below the null threshold (266.4° for 21 sessions), demonstrating remarkable stability." This definition of PD stability is rather problematic, since it allows a neuron to shift its tuning to the opposite direction before being considered “unstable”. Circular statistic tests (perhaps the Rayleigh test) that measure the degree of directional concentration would be more appropriate to assess stability.

We appreciate the reviewer’s thoughtful comment and agree that a large absolute shift in preferred direction (PD) may appear inconsistent with the notion of stability. Our analysis, however, evaluates stability in a relative rather than absolute sense. Specifically, we compare each neuron’s observed PD span to a null distribution obtained from simulated PD trajectories with randomly permuted directions across sessions. A neuron is considered “stable” only if its PD variation is significantly smaller than what would be expected from random drift across the same number of days.

This approach captures whether a neuron changes less than chance rather than whether its PD remains near a fixed angle. We recognize that this may not have been clearly articulated in the original text and have revised the manuscript accordingly. We also appreciate the reviewer’s suggestion regarding circular statistical tests such as the Rayleigh test. While these tests assess concentration around a mean direction, they are less suited for scenarios where the mean direction itself may shift gradually over long timescales, which is a main feature of this study.

**References**
1.	E. H. Van Beest, C. Bimbard, J. M. J. Fabre, S. W. Dodgson, F. Takács, P. Coen, A. Lebedeva, K. D. Harris, M. Carandini, Tracking neurons across days with high-density probes. Nat Methods 22, 778–787 (2024).

2.	Enny van Beest, Celian Bimbard, Julie Fabre, Flora Takacs, Philip Coen, Anna Lebedeva, Kenneth Harris, Matteo Carandini, UnitMatch Demo - data, (2023); https://doi.org/10.6084/m9.figshare.24305758.v1.

3.	M. Pachitariu, S. Sridhar, J. Pennington, C. Stringer, Spike sorting with Kilosort4. Nat Methods 21, 914–921 (2024).

4.	P. Yger, G. L. Spampinato, E. Esposito, B. Lefebvre, S. Deny, C. Gardella, M. Stimberg, F. Jetter, G. Zeck, S. Picaud, J. Duebel, O. Marre, A spike sorting toolbox for up to thousands of electrodes validated with ground truth recordings in vitro and in vivo. eLife 7, e34518 (2018).

5.	J. E. Chung, J. F. Magland, A. H. Barnett, V. M. Tolosa, A. C. Tooker, K. Y. Lee, K. G. Shah, S. H. Felix, L. M. Frank, L. F. Greengard, A Fully Automated Approach to Spike Sorting. Neuron 95, 1381-1394.e6 (2017).

6.	G. Hilgen, M. Sorbaro, S. Pirmoradian, J.-O. Muthmann, I. E. Kepiro, S. Ullo, C. J. Ramirez, A. Puente Encinas, A. Maccione, L. Berdondini, V. Murino, D. Sona, F. Cella Zanacchi, E. Sernagor, M. H. Hennig, Unsupervised Spike Sorting for Large-Scale, High-Density Multielectrode Arrays. Cell Reports 18, 2521–2532 (2017).

7.	A. K. Dhawale, R. Poddar, S. B. Wolff, V. A. Normand, E. Kopelowitz, B. P. Ölveczky, Automated long-term recording and analysis of neural activity in behaving animals. eLife 6, e27702 (2017).

8.	K. Ganguly, J. M. Carmena, Emergence of a Stable Cortical Map for Neuroprosthetic Control. PLoS Biol 7, e1000153 (2009).

9.	K. Ganguly, D. F. Dimitrov, J. D. Wallis, J. M. Carmena, Reversible large-scale modification of cortical networks during neuroprosthetic control. Nat Neurosci 14, 662–667 (2011).

10.	P. T. Sadtler, K. M. Quick, M. D. Golub, S. M. Chase, S. I. Ryu, E. C. Tyler-Kabara, B. M. Yu, A. P. Batista, Neural constraints on learning. Nature 512, 423–426 (2014).

11.	E. R. Oby, M. D. Golub, J. A. Hennig, A. D. Degenhart, E. C. Tyler-Kabara, B. M. Yu, S. M. Chase, A. P. Batista, New neural activity patterns emerge with long-term learning. Proceedings of the National Academy of Sciences 116, 15210–15215 (2019).
