# Supplementary material for: Long-term neuron tracking reveals balance of stability and plasticity in functional properties
Source: PLoS One. 2026 Jun 29;21(6):e0321830. doi: 10.1371/journal.pone.0321830 (PMC13313350; doi:10.1371/journal.pone.0321830)
Supplement: Peer review history

# Peer review: Decision Letter 0

**DOI:** 10.1371/journal.pone.0321830.r001

**Authors:** Abdolvahed Narmashiri

5 Nov 2025

PONE-D-25-13192 Long-term neuron tracking reveals balance of stability and plasticity in functional properties PLOS ONE

Dear Dr. Santacruz,

Thank you for submitting your manuscript to PLOS ONE. After careful consideration, we feel that it has merit but does not fully meet PLOS ONE’s publication criteria as it currently stands. Therefore, we invite you to submit a revised version of the manuscript that addresses the points raised during the review process.

Please submit your revised manuscript by Dec 20 2025 11:59PM. If you will need more time than this to complete your revisions, please reply to this message or contact the journal office at plosone@plos.org. When you're ready to submit your revision, log on to https://www.editorialmanager.com/pone/ and select the 'Submissions Needing Revision' folder to locate your manuscript file.

Please include the following items when submitting your revised manuscript:

- A rebuttal letter that responds to each point raised by the academic editor and reviewer(s). You should upload this letter as a separate file labeled 'Response to Reviewers'.
- A marked-up copy of your manuscript that highlights changes made to the original version. You should upload this as a separate file labeled 'Revised Manuscript with Track Changes'.
- An unmarked version of your revised paper without tracked changes. You should upload this as a separate file labeled 'Manuscript'.

If you would like to make changes to your financial disclosure, please include your updated statement in your cover letter. Guidelines for resubmitting your figure files are available below the reviewer comments at the end of this letter.

If applicable, we recommend that you deposit your laboratory protocols in protocols.io to enhance the reproducibility of your results. Protocols.io assigns your protocol its own identifier (DOI) so that it can be cited independently in the future. For instructions see: https://journals.plos.org/plosone/s/submission-guidelines#loc-laboratory-protocols. Additionally, PLOS ONE offers an option for publishing peer-reviewed Lab Protocol articles, which describe protocols hosted on protocols.io. Read more information on sharing protocols at https://plos.org/protocols?utm_medium=editorial-email&utm_source=authorletters&utm_campaign=protocols.

We look forward to receiving your revised manuscript.

Kind regards,

Abdolvahed Narmashiri

Academic Editor

PLOS ONE

Journal Requirements:

When submitting your revision, we need you to address these additional requirements.

1. Please ensure that your manuscript meets PLOS ONE's style requirements, including those for file naming. The PLOS ONE style templates can be found at

https://journals.plos.org/plosone/s/file?id=wjVg/PLOSOne_formatting_sample_main_body.pdf and

https://journals.plos.org/plosone/s/file?id=ba62/PLOSOne_formatting_sample_title_authors_affiliations.pdf

2. PLOS requires an ORCID iD for the corresponding author in Editorial Manager on papers submitted after December 6th, 2016. Please ensure that you have an ORCID iD and that it is validated in Editorial Manager. To do this, go to ‘Update my Information’ (in the upper left-hand corner of the main menu), and click on the Fetch/Validate link next to the ORCID field. This will take you to the ORCID site and allow you to create a new iD or authenticate a pre-existing iD in Editorial Manager.

3. Thank you for stating the following financial disclosure:

This work was supported by Whitehall Foundation [2022-12-071] and National Science Foundation CAREER Award [2145412].

Please state what role the funders took in the study. If the funders had no role, please state: "The funders had no role in study design, data collection and analysis, decision to publish, or preparation of the manuscript."

If this statement is not correct you must amend it as needed.

Please include this amended Role of Funder statement in your cover letter; we will change the online submission form on your behalf.

4. Thank you for stating the following in the Acknowledgments Section of your manuscript:

This work was supported by Whitehall Foundation [2022-12-071] and National Science Foundation CAREER Award [2145412].

We note that you have provided funding information that is not currently declared in your Funding Statement. However, funding information should not appear in the Acknowledgments section or other areas of your manuscript. We will only publish funding information present in the Funding Statement section of the online submission form.

Please remove any funding-related text from the manuscript and let us know how you would like to update your Funding Statement. Currently, your Funding Statement reads as follows:

This work was supported by Whitehall Foundation [2022-12-071] and National Science Foundation CAREER Award [2145412].

Please include your amended statements within your cover letter; we will change the online submission form on your behalf.

5. We note that you have indicated that there are restrictions to data sharing for this study. PLOS only allows data to be available upon request if there are legal or ethical restrictions on sharing data publicly. For more information on unacceptable data access restrictions, please see http://journals.plos.org/plosone/s/data-availability#loc-unacceptable-data-access-restrictions.

Before we proceed with your manuscript, please address the following prompts:

a) If there are ethical or legal restrictions on sharing a de-identified data set, please explain them in detail (e.g., data contain potentially identifying or sensitive patient information, data are owned by a third-party organization, etc.) and who has imposed them (e.g., a Research Ethics Committee or Institutional Review Board, etc.). Please also provide contact information for a data access committee, ethics committee, or other institutional body to which data requests may be sent.

b) If there are no restrictions, please upload the minimal anonymized data set necessary to replicate your study findings to a stable, public repository and provide us with the relevant URLs, DOIs, or accession numbers. For a list of recommended repositories, please see

https://journals.plos.org/plosone/s/recommended-repositories. You also have the option of uploading the data as Supporting Information files, but we would recommend depositing data directly to a data repository if possible.

We will update your Data Availability statement on your behalf to reflect the information you provide.

6. When completing the data availability statement of the submission form, you indicated that you will make your data available on acceptance. We strongly recommend all authors decide on a data sharing plan before acceptance, as the process can be lengthy and hold up publication timelines. Please note that, though access restrictions are acceptable now, your entire data will need to be made freely accessible if your manuscript is accepted for publication. This policy applies to all data except where public deposition would breach compliance with the protocol approved by your research ethics board. If you are unable to adhere to our open data policy, please kindly revise your statement to explain your reasoning and we will seek the editor's input on an exemption. Please be assured that, once you have provided your new statement, the assessment of your exemption will not hold up the peer review process.

7. If the reviewer comments include a recommendation to cite specific previously published works, please review and evaluate these publications to determine whether they are relevant and should be cited. There is no requirement to cite these works unless the editor has indicated otherwise.

[Note: HTML markup is below. Please do not edit.]

Reviewers' comments:

Reviewer's Responses to Questions

**Comments to the Author**

1. Is the manuscript technically sound, and do the data support the conclusions?

The manuscript must describe a technically sound piece of scientific research with data that supports the conclusions. Experiments must have been conducted rigorously, with appropriate controls, replication, and sample sizes. The conclusions must be drawn appropriately based on the data presented.

Reviewer #1: Yes

Reviewer #2: Partly

Reviewer #3: Yes

**********

2. Has the statistical analysis been performed appropriately and rigorously?

Reviewer #1: Yes

Reviewer #2: No

Reviewer #3: Yes

**********

3. Have the authors made all data underlying the findings in their manuscript fully available?

The PLOS Data policy requires authors to make all data underlying the findings described in their manuscript fully available without restriction, with rare exception (please refer to the Data Availability Statement in the manuscript PDF file). The data should be provided as part of the manuscript or its supporting information, or deposited to a public repository. For example, in addition to summary statistics, the data points behind means, medians and variance measures should be available. If there are restrictions on publicly sharing data—e.g. participant privacy or use of data from a third party—those must be specified.

Reviewer #1: Yes

Reviewer #2: No

Reviewer #3: No

**********

4. Is the manuscript presented in an intelligible fashion and written in standard English?

PLOS ONE does not copyedit accepted manuscripts, so the language in submitted articles must be clear, correct, and unambiguous. Any typographical or grammatical errors should be corrected at revision, so please note any specific errors here.

Reviewer #1: Yes

Reviewer #2: Yes

Reviewer #3: Yes

**********

5. Review Comments to the Author

Please use the space provided to explain your answers to the questions above. You may also include additional comments for the author, including concerns about dual publication, research ethics, or publication ethics. (Please upload your review as an attachment if it exceeds 20,000 characters)

Reviewer #1: This is an automated report for PONE-D-25-13192. This report was solicited by the PLOS One editorial team and provided by ScreenIT.

ScreenIT is an independent group of scientists developing automated tools that analyze academic papers. A set of automated tools screened your submitted manuscript and provided the report below. Each tool was created by your academic colleagues with the goal of helping authors. The tools look for factors that are important for transparency, rigor and reproducibility, and we hope that the report might help you to improve reporting in your manuscript. Within the report you will find links to more information about the items that the tools check. These links include helpful papers, websites, or videos that explain why the item is important. While our screening tools aim to improve and maintain quality standards they may, on occasion, miss nuances specific to your study type or flag something incorrectly. Each tool has limitations that are described on the ScreenIT website. The tools screen the main file for the paper; they are not able to screen supplements stored in separate files. Please note that the Academic Editor had access to these comments while making a decision on your manuscript. The Academic Editor may ask that issues flagged in this report be addressed. If you would like to provide feedback on the ScreenIT tool, please email the team at ScreenIt@bih-charite.de. If you have questions or concerns about the review process, please contact the PLOS One office at plosone@plos.org.

Reviewer #2: In this manuscript the authors report a method to track single-unit waveforms recorded from classic MEAs across sessions and days. They start with neurons that were ‘online’ sorted by their recording system within a session and use a waveform similarity metric to track these sorted neurons across days/sessions.

The underlying algorithm is relatively simple, it’s a pairwise comparison of mean neural waveforms. Specifically, it computes the euclidean distance and the pearson correlation, normalizes it, and takes the mean of these two metrics. The similarity is compared to a null-distribution and if it is significant the neural waveforms from different days are ‘merged’. Using this method, they can track ~40% of their total sorted neurons across multiple days. The median is close to ~20 days, but a subset were tracked over ~100 days.

For these tracked neurons, they analyze the mean firing-rates, inter-spike-intervals, tuning direction and field coupling and state that these properties are mostly stable (although see comments).

The major strengths of this paper are the long-term neural recordings, and the manuscript is largely straightforward and the methods reported here could be useful to researchers using classic MEA probes.

The main weakness of the paper is that the lack of comparisons to other methods make it hard to determine how well the method works, and the biological insight gained from these analyses is very limited, and although the results here are clear - they could use some more analyses, nuance and controls.

Major Comments:

1. How good is this method compared to other approaches? The lack of a ‘ground truth’ makes it difficult to evaluate how much better this method is or how effective it is. A meaningful comparison to other methods - i.e. ones that use other features or different metrics would make the effectiveness of this system more obvious and a clear validation step would be useful.

2. The system seems to heavily rely on online-sorted/pre-sorted neurons, which can have their own error rates etc - however, more modern tools combine sorting and tracking these steps into a single computation, which allows them to capture drift over the session - could the authors comment on why they chose their approach?

3. For the more biologically relevant results reported here, some more comparative analysis is warranted. The role of the task in the results reported here is largely ignored - There are two kinds of neurons in these recordings, neurons that are used for the kalman filter and presumably other unrelated neurons - which wouldn’t have a pd. A comparative analysis for these two kinds of neurons would have been a useful discovery - are the neurons that are task related the more stable ones? And the ones which are not task related are the ones that go silent or have larger changes? Are there any consistent trends about which neurons shift and which dont? Is it related to task performance, does stability depend on how informative that neural response was for the kalman filter?

4. I am having a hard time understanding how one can claim stability when the PD for a neuron changes over 200 degrees over time. This shows stability in the neural recording, not in the neural responses. For determining PDs did the authors use a threshold for finding neurons that had a good fit to the cosine model? The shifting PDs could also be a sign of assigning PDs to neurons that aren’t actually directionally tuned.

5. Was there any behavioral consequence for having stable neurons? Right now this is largely a phenomenological study, it could help if the authors had a biological hypothesis this allowed one to test.

Minor comments:

1. Its not clear to me why you would use the mean of the pearson correlation and the euclidean distance for similarity scores, instead of treating them as a 2D vector - as the the authors themselves point out, these capture two different aspects of similarity

2. The reasoning for comparison of mean firing rates across sessions was not clear to me, wouldn’t comparing only the first and last sessions, bias your estimates toward stability since many neurons are recorded for <20 days? Was there a measurement of session by session mean firing rate changes, maybe a regression analysis here would clarify how the number of sessions alters firing rates?

3. In general, readability would have been improved if the results section used more declarative titles that described the result instead of a generic title. For e.g. instead of titles like “Neuron tracking”, something more along the lines of “Neurons were tracked for xx days” etc.

4. The copy I received for review had the figure legends inserted at odd locations into the main text, please check that the submission is appropriately formatted before sending it out to review.

Specific text suggestions:

“Neural stability is essential for executing learned … “

While I understand the general sentiment behind this statement, this dichotomy is not true - Degeneracy in neural circuits enables skilled behavior even though neural firing patterns are not identical. E.g. EMG activity (and therefore motor neuron activity) can vary substantially, but the hand kinematics can be identical. For cortical studies, the Null-Space shown by kaufman and colleagues is another example of neural acitivity in individual neurons not being deterministic for behavior. There are more examples for other flexible behaviors such as the STG patterns of neural activity shown by Prinz and Marder and in other invertebrates. It would be prudent to dial back these dichotomies.

For stable/unstable representations since the authors cited the Liberti et al 2016 paper, they should also cite the Katlowitz et al 2018 neuron paper which challenges the observations in the Liberti paper.

Reviewer #3: In the present study, the authors implemented a novel methodology to track neural activity across recording sessions spread over many days (3 months), with the larger goal of assessing the long term stability of neural activity associated with the control of a robotic arm using a Brain-Machine Interface (BMI) which translated neural population spiking activity into cursor velocity, enabling macaques to perform a "center-out" task. Using their tracking approach, based in waveform similarity comparison across sessions, the authors were able to successfully track individual neurons across days and to assess the stability of their functional properties (direction tuning, Inter-Spike Interval distribution, and Spike-Field Coherence), and show that individual neurons exhibit a high degree of stability, while some plasticity is present, and hypothesize that the balance between stability and flexibility allow motor learning.

The manuscript is nicely and clearly written, and presents the information in a clear and concise manner. The data analysis is sound, and the authors utilized stringent criteria to select those neurons that would be considerer to be the same across different recording sessions, showing that their intrinsic functional properties were preserved.

The main weakness of the manuscript is the lack of some sort of "ground truth" test. I believe it would be relatively easy to generate synthetic data that could be analyzed using the proposed tracking method. This would allow to also determine what kind of variations in the detected spikes may lead the tracking algorithm to fail. Another option would be to apply the tracking method to subsets of data taking randomly a fraction of the trials on each session, and assessing the consistency of the results.

Other than that, I only have a few minor comments, which I list below:

1) Please specify what kind of tungsten electrode arrays were used (i.e. manufacturer) and their characteristics (tip length, spacing, impedance range, etc.)

2) How stable were the units sorted during a given recording session. It would be useful for the reader to have an idea of the waveform stability. The authors mention that only units with high SNR were used for the analysis, it would be nice to know how stable the SNR was during a session, in addition to across sessions.

3) It would be useful to indicate in Figure 1 that the log transformed Euclidean distance was used in the similarity matrix, to avoid confusion in the readers that may be thinking of correlation as "similarity" vs distance as "dissimilarity".

4) Line 440. "Monkey B exhibited significant changes in mean firing rates, whereas Monkey A did not." The distributions in Figure 2C look very similar. In fact, the distribution of relative changes in mean FR for monkey B peaks at zero, while the distributor monkey A peaks below 0. Therefore, this conclusion is hard to accept, at least without a statistic. Statistical test results are given for peak and through amplitude, but not for mean FR.

5) Lines 481-484. "An example tracked neuron (Fig 4C) exhibited a PD around 482 37.6°, gradually shifting clockwise to 246.8° over 91 days, yet its PD span (150.8°) 483 remained well below the null threshold (266.4° for 21 sessions), demonstrating 484 remarkable stability." This definition of PD stability is rather problematic, since it allows a neuron to shift its tuning to the opposite direction before being considered “unstable”. Circular statistic tests (perhaps the Rayleigh test) that measure the degree of directional concentration would be more appropriate to assess stability.

6) Related to comment 5. Lines 562-564 "We demonstrated that spiking dynamics, directional 563 tuning, and spike-field interactions remained stable over time a large proportion of 564 neurons, while the remaining neurons were more plastic". Sure, for the short term (neighboring sessions) this is true, but I'd argue it is debatable for the long term, given the authors’ definition of stability.

**********

6. PLOS authors have the option to publish the peer review history of their article (what does this mean?). If published, this will include your full peer review and any attached files.

If you choose “no”, your identity will remain anonymous but your review may still be made public.

**Do you want your identity to be public for this peer review?** For information about this choice, including consent withdrawal, please see our Privacy Policy.

Reviewer #1: No

Reviewer #2: No

Reviewer #3: No

**********

[NOTE: If reviewer comments were submitted as an attachment file, they will be attached to this email and accessible via the submission site. Please log into your account, locate the manuscript record, and check for the action link "View Attachments". If this link does not appear, there are no attachment files.]

To ensure your figures meet our technical requirements, please review our figure guidelines: https://journals.plos.org/plosone/s/figures

You may also use PLOS’s free figure tool, NAAS, to help you prepare publication quality figures: https://journals.plos.org/plosone/s/figures#loc-tools-for-figure-preparation.

NAAS will assess whether your figures meet our technical requirements by comparing each figure against our figure specifications.

Attachment Submitted filename: report_10.1101+2025.03.14.643204.pdf

# Author comment: Author response to Decision Letter 1

**DOI:** 10.1371/journal.pone.0321830.r002

5 May 2026

We sincerely thank the editors and reviewers for their detailed feedback and suggestions on our manuscript. We are honored that Reviewer #2 believes that “the manuscript is largely straightforward and the methods reported here could be useful to researchers using classic MEA probes” and Reviewer #3 believes that “The manuscript is nicely and clearly written and presents the information in a clear and concise manner. The data analysis is sound, and the authors utilized stringent criteria to select those neurons that would be considered to be the same across different recording sessions, showing that their intrinsic functional properties were preserved”. We have made substantial revisions to the manuscript that incorporate all the information and additional material required by the reviewers. The revision has greatly improved the quality of the paper and clarified our core concepts. Below we have addressed each of the reviewers’ comments individually and highlighted where corresponding changes have been made in the manuscript.

Reviewer #2:

In this manuscript the authors report a method to track single-unit waveforms recorded from classic MEAs across sessions and days. They start with neurons that were ‘online’ sorted by their recording system within a session and use a waveform similarity metric to track these sorted neurons across days/sessions. The underlying algorithm is relatively simple, it’s a pairwise comparison of mean neural waveforms. Specifically, it computes the euclidean distance and the pearson correlation, normalizes it, and takes the mean of these two metrics. The similarity is compared to a null-distribution and if it is significant the neural waveforms from different days are ‘merged’. Using this method, they can track ~40% of their total sorted neurons across multiple days. The median is close to ~20 days, but a subset were tracked over ~100 days. For these tracked neurons, they analyze the mean firing-rates, inter-spike-intervals, tuning direction and field coupling and state that these properties are mostly stable (although see comments). The major strengths of this paper are the long-term neural recordings, and the manuscript is largely straightforward and the methods reported here could be useful to researchers using classic MEA probes. The main weakness of the paper is that the lack of comparisons to other methods make it hard to determine how well the method works, and the biological insight gained from these analyses is very limited, and although the results here are clear - they could use some more analyses, nuance and controls.

We thank the reviewer for appreciating this manuscript and giving strong feedback to strengthen the paper. We have revised the manuscript accordingly.

Major comments:

1.How good is this method compared to other approaches? The lack of a ‘ground truth’ makes it difficult to evaluate how much better this method is or how effective it is. A meaningful comparison to other methods - i.e. ones that use other features or different metrics would make the effectiveness of this system more obvious and a clear validation step would be useful.

We thank the reviewer for the excellent suggestion to benchmark our approach against existing methods. To address this, we directly compared our algorithm with UnitMatch (1), a well-regarded method designed specifically for high-density probes such as Neuropixels. Importantly, UnitMatch provides a publicly available dataset (2), allowing a transparent and reproducible head-to-head evaluation.

Although both approaches leverage waveform similarity to identify neurons across sessions, they were developed for fundamentally different recording regimes. UnitMatch is optimized for high-density arrays, where individual neurons are simultaneously detected across multiple nearby channels. This enables the use of spatial metrics such as decay similarity, centroid position, and trajectory that are inherently unavailable in the single- or low-channel recordings for which our algorithm is designed. Thus, the comparison is not meant to determine superiority, but rather to clarify use cases and highlight complementary strengths.

In the UnitMatch dataset, five rodents were implanted with chronic Neuropixels probes, each with two sessions collected one day apart. Besides the original UnitMatch procedure, we applied our algorithm to the same data. Across subjects, our method recovered many within-day matches, suggesting that it generalizes well even to data outside its intended domain. As expected for an approach that does not use spatial information, our sensitivity (0.144 ± 0.042) was lower than UnitMatch’s (0.454 ± 0.009). However, our specificity remained perfect (1.0000 ± 0.0000), matching UnitMatch’s performance (0.9992 ± 0.0005).

We view this result positively and believe it reflects deliberate design choices rather than a limitation. First, our method prioritizes high specificity, which is essential for longitudinal tracking: false positives have far more damaging consequences than missed matches, particularly in clinical or multi-session cognitive studies. Second, the benchmark dataset contains only two sessions per subject and therefore does not capture the temporal variability across months-long datasets that our algorithm was built for. Third, because UnitMatch’s decisions rely heavily on spatial features unavailable in our data domain, the sensitivity difference primarily reflects sensor constraints rather than algorithmic weakness. Finally, UnitMatch provides a useful benchmark but not a definitive ground truth. Some matches labeled as correct by UnitMatch may be overly liberal, meaning our algorithm’s “misses” may in fact represent correct rejections.

Overall, this comparison demonstrates that our method maintains excellent specificity and produces reasonable matches even in a dataset optimized for a different type of electrode array. This supports the robustness of our approach and clarifies the scenarios in which it provides advantages: long-term, multi-session studies with conventional MEAs where spatial features are not available. We have included this in the Results section.

2. The system seems to heavily rely on online-sorted/pre-sorted neurons, which can have their own error rates etc - however, more modern tools combine sorting and tracking these steps into a single computation, which allows them to capture drift over the session - could the authors comment on why they chose their approach?

We thank the reviewer for this critical feedback. Indeed, there are plenty of tools that combine sorting and tracking. Indeed, several modern spike sorting frameworks do combine sorting and drift tracking into a single computational procedure. Examples include Kilosort (3), SpyKING CIRCUS 2.0 (4), MountainSort 4 (5), and Herdingspikes2 (6), all of which incorporate template evolution or explicit spatial drift correction during the sorting process. While these tools jointly perform spike sorting and drift correction, they are designed primarily for within-session drift and rely heavily on high-density spatial information, such as that provided by Neuropixels probes. These approaches solve a different problem from the one addressed in our work.

First, our goal is to identify neuron identity across sessions and across days, not just track sub-second or minute-scale drift within a single session. Most of these sorters, such as Kilosort or FAST (7), assume temporal continuity: templates evolve gradually during a single uninterrupted recording. However, they do not infer correspondence across discontinuous sessions, where electrodes may have been reconnected, tissue may have shifted substantially, and waveform appearance may change abruptly.

Second, drift-correcting sorters depend on spatial features that are unavailable in conventional MEAs, such as classic microwire arrays, Utah arrays, or tetrode-based systems. These settings were operated by many laboratories, including ours. Tools like Kilosort or SpyKING CIRCUS leverage information such as depth-based template drift, multi-channel spatial footprint, and consistent relative channel amplitudes. In low-density systems, each neuron appears on only one channel, making these methods unsuitable for cross-day identity tracking in such datasets.

Third, even modern joint-sorting approaches do not guarantee cross-day “identity stability.” Their merging decisions are typically optimized for accurate sorting within a recording session. Without explicit cross-session modeling, the same biological neuron recorded across different days will usually be labeled as different units, despite good sorting quality.

Finally, our algorithm is designed for the challenges inherent to multi-session, multi-day chronic recordings, such as those encountered in human intracranial recordings or long-term animal experiments. Our focus is on developing a consistent and interpretable metric space in which units from different days can be compared even when the waveform shapes differ due to biological or recording-related changes. This differs fundamentally from real-time drift correction and complements, rather than replaces, existing sort-and-track methods.

Taken together, while joint sorting-tracking tools have advanced within-session accuracy, they do not address the key question targeted by our work: how to robustly identify neurons across discontinuous recording sessions using conventional electrode arrays. Our approach directly tackles this gap.

3. For the more biologically relevant results reported here, some more comparative analysis is warranted. The role of the task in the results reported here is largely ignored - There are two kinds of neurons in these recordings, neurons that are used for the kalman filter and presumably other unrelated neurons - which wouldn’t have a pd. A comparative analysis for these two kinds of neurons would have been a useful discovery - are the neurons that are task related the more stable ones? And the ones which are not task related are the ones that go silent or have larger changes? Are there any consistent trends about which neurons shift and which dont? Is it related to task performance, does stability depend on how informative that neural response was for the kalman filter?

We thank the reviewer for this thoughtful suggestion. We agree that understanding how task involvement relates to tuning stability is biologically meaningful. However, in our dataset, most tracked units were incorporated into the Kalman decoder at least some of the time (e.g., 72/114 for Monkey A; 293/353 for Monkey B). As a result, a strict binary comparison between “task-related” and “task-unrelated” neurons is not feasible because very few neurons were never used.

To address the reviewer’s concern, we instead quantified task involvement continuously, using the fraction of sessions in which each neuron contributed to the Kalman filter. This metric captures the degree to which a neuron participated in task control across days. We then compared this measure with each neuron’s PD span.

Across animals, we found weak to moderate relationships (Spearman ρ = 0.32 in Monkey A; ρ = 0.59 in Monkey B), indicating that neurons used more frequently in the decoder tend to have slightly smaller PD spans, but that task usage explains only a modest portion of the variance. This result suggests that tuning stability is not exclusively a property of the “direct” population.

Importantly, extensive BMI literature shows that neurons not explicitly used in the decoder (“indirect neurons”) nonetheless develop task-related modulation and exhibit significant tuning changes during learning. This phenomenon has been demonstrated in classic work showing network-wide plasticity (8, 9), population-dynamics studies showing adaptation across the entire manifold (10), and long-term learning studies documenting reorganization in both direct and indirect units (11). These findings corroborate our observation that neurons outside the decoder can still exhibit meaningful directional tuning and stability.

4. I am having a hard time understanding how one can claim stability when the PD for a neuron changes over 200 degrees over time. This shows stability in the neural recording, not in the neural responses. For determining PDs did the authors use a threshold for finding neurons that had a good fit to the cosine model? The shifting PDs could also be a sign of assigning PDs to neurons that aren’t actually directionally tuned.

We thank the reviewer for raising this point and appreciate the opportunity to clarify our definition of PD stability. We are not claiming that a neuron with a 200° change in preferred direction (PD) is “stable” in an absolute sense. Instead, stability in our manuscript is defined relative to a null distribution generated from random PD trajectories over the same duration. The PD span therefore reflects whether a neuron’s directional preference changes less than expected by chance, not whether the absolute magnitude of its change is small. This distinction may not have been sufficiently clear in the original text, and we have now revised the manuscript to emphasize this point.

Regarding the concern about directional tuning, we fully agree that PD estimates are only meaningful when the cosine model provides a reliable fit. To quantify tuning quality, we computed bootstrap confidence intervals (CIs) for each neuron’s PD. Poorly tuned neurons exhibit large PD CIs due to high uncertainty in the cosine fit. Across the population, we found that PD estimates were generally reliable: the median 95% CI widths were 58.43° for Monkey A and 33.36° for Monkey B, indicating good fits overall.

To ensure that downstream analyses were based only on well-tuned units, we excluded neurons with PD CI widths greater than 120°. This threshold removed 224 units in Monkey A (34.91%) and 375 units in Monkey B (22.81%). We regenerated the results in Figure 4 using only these well-tuned neurons (Reviewer Response Figure 1) as well as Figure 7 (Reviewer Response Figure 2). We found that all major conclusions remained unchanged. Applying this criterion therefore strengthens our analysis by ensuring that neurons with unreliable directional tuning do not influence PD stability estimates.

Reviewer Response Figure 1. Revised Figure 4 in the manuscript. (A) The changes in PDs between neighboring sessions in tracked neurons (Monkey A: N=316, p=0.329; Monkey B: N=1045, p=0.75. One-sample t-test). (B) The numbers and percentages of tracked neurons that had stable and unstable PDs, as characterized by the PD span. (C) The PDs and their corresponding mean waveforms of an example tracked neuron.

Reviewer Response Figure 2. Revised Figure 7 in the manuscript. The Venn diagrams showed the numbers of tracked neurons in each factorial combination of the stability of ISI, PD, and SFC. Almost all of tracked neurons were stable in at least one functional property (107 out of 116 tracked neurons in Monkey A and 323 out of 354 tracked neurons in Monkey B), whereas more than 30% of tracked neurons have stable ISI, PD, and SFC (25% for Monkey A and 32% for Monkey B).

5. Was there any behavioral consequence for having stable neurons? Right now this is largely a phenomenological study, it could help if the authors had a biological hypothesis this allowed one to test.

We thank the reviewer for raising this important conceptual point. Our biological hypothesis in this work is that individual neurons exhibit quantifiable stability (or instability) in their preferred direction over long timescales, and that such stability can be rigorously measured using our tracking framework. Thus, the primary goal of the study is to characterize the structure of PD stability across months of intracortical recordings rather than to manipulate stability to test its behavioral consequences.

Our current dataset does not permit a causal analysis of how stable versus unstable neurons influence behavior because (a) the decoder used almost all available units (see Reviewer #2, Major comment 3), leaving no experimental manipulation of which neurons contributed to control, and (b) neural stability can only be assessed retrospectively after many sessions have accrued. Thus, behavioral differences conditioned on neuron stability cannot b

Attachment Submitted filename: NT - R1 - point-by-point - final.docx

# Peer review: Decision Letter 1

**DOI:** 10.1371/journal.pone.0321830.r003

**Authors:** Abdolvahed Narmashiri

10 Jun 2026

Long-term neuron tracking reveals balance of stability and plasticity in functional properties

PONE-D-25-13192R1

Dear Dr. Santacruz,

We’re pleased to inform you that your manuscript has been judged scientifically suitable for publication and will be formally accepted for publication once it meets all outstanding technical requirements.

Within one week, you’ll receive an e-mail detailing the required amendments. When these have been addressed, you’ll receive a formal acceptance letter and your manuscript will be scheduled for publication.

An invoice will be generated when your article is formally accepted. Please note, if your institution has a publishing partnership with PLOS and your article meets the relevant criteria, all or part of your publication costs will be covered. Please make sure your user information is up-to-date by logging into Editorial Manager at Editorial Manager® and clicking the ‘Update My Information' link at the top of the page. For questions related to billing, please contact billing support.

If your institution or institutions have a press office, please notify them about your upcoming paper to help maximize its impact. If they’ll be preparing press materials, please inform our press team as soon as possible -- no later than 48 hours after receiving the formal acceptance. Your manuscript will remain under strict press embargo until 2 pm Eastern Time on the date of publication. For more information, please contact onepress@plos.org.

Kind regards,

Abdolvahed Narmashiri

Academic Editor

PLOS One

Additional Editor Comments (optional):

Reviewers' comments:

Reviewer's Responses to Questions

**Comments to the Author**

1. If the authors have adequately addressed your comments raised in a previous round of review and you feel that this manuscript is now acceptable for publication, you may indicate that here to bypass the “Comments to the Author” section, enter your conflict of interest statement in the “Confidential to Editor” section, and submit your "Accept" recommendation.

Reviewer #2: All comments have been addressed

Reviewer #3: All comments have been addressed

**********

2. Is the manuscript technically sound, and do the data support the conclusions?

The manuscript must describe a technically sound piece of scientific research with data that supports the conclusions. Experiments must have been conducted rigorously, with appropriate controls, replication, and sample sizes. The conclusions must be drawn appropriately based on the data presented.

Reviewer #2: Yes

Reviewer #3: Yes

**********

3. Has the statistical analysis been performed appropriately and rigorously?

Reviewer #2: Yes

Reviewer #3: Yes

**********

4. Have the authors made all data underlying the findings in their manuscript fully available?

The PLOS Data policy requires authors to make all data underlying the findings described in their manuscript fully available without restriction, with rare exception (please refer to the Data Availability Statement in the manuscript PDF file). The data should be provided as part of the manuscript or its supporting information, or deposited to a public repository. For example, in addition to summary statistics, the data points behind means, medians and variance measures should be available. If there are restrictions on publicly sharing data—e.g. participant privacy or use of data from a third party—those must be specified.

Reviewer #2: No

Reviewer #3: Yes

**********

5. Is the manuscript presented in an intelligible fashion and written in standard English?

PLOS ONE does not copyedit accepted manuscripts, so the language in submitted articles must be clear, correct, and unambiguous. Any typographical or grammatical errors should be corrected at revision, so please note any specific errors here.

Reviewer #2: Yes

Reviewer #3: Yes

**********

6. Review Comments to the Author

Please use the space provided to explain your answers to the questions above. You may also include additional comments for the author, including concerns about dual publication, research ethics, or publication ethics. (Please upload your review as an attachment if it exceeds 20,000 characters)

Reviewer #2: (No Response)

Reviewer #3: The authors have addressed my comments.

I have no further concerns.

**********

7. PLOS authors have the option to publish the peer review history of their article (what does this mean?). If published, this will include your full peer review and any attached files.

If you choose “no”, your identity will remain anonymous but your review may still be made public.

**Do you want your identity to be public for this peer review?** For information about this choice, including consent withdrawal, please see our Privacy Policy.

Reviewer #2: No

Reviewer #3: No

**********

# Editor report: Acceptance letter

**DOI:** 10.1371/journal.pone.0321830.r004

**Authors:** Abdolvahed Narmashiri

PONE-D-25-13192R1

PLOS One

Dear Dr. Santacruz,

I'm pleased to inform you that your manuscript has been deemed suitable for publication in PLOS One. Congratulations! Your manuscript is now being handed over to our production team.

At this stage, our production department will prepare your paper for publication. This includes ensuring the following:

* All references, tables, and figures are properly cited

* All relevant supporting information is included in the manuscript submission,

* There are no issues that prevent the paper from being properly typeset

You will receive further instructions from the production team, including instructions on how to review your proof when it is ready. Please keep in mind that we are working through a large volume of accepted articles, so please give us a few days to review your paper and let you know the next and final steps.

Lastly, if your institution or institutions have a press office, please let them know about your upcoming paper now to help maximize its impact. If they'll be preparing press materials, please inform our press team within the next 48 hours. Your manuscript will remain under strict press embargo until 2 pm Eastern Time on the date of publication. For more information, please contact onepress@plos.org.

You will receive an invoice from PLOS for your publication fee after your manuscript has reached the completed accept phase. If you receive an email requesting payment before acceptance or for any other service, this may be a phishing scheme. Learn how to identify phishing emails and protect your accounts at https://explore.plos.org/phishing.

If we can help with anything else, please email us at customercare@plos.org.

Thank you for submitting your work to PLOS ONE and supporting open access.

Kind regards,

PLOS ONE Editorial Office Staff

on behalf of

Dr. Abdolvahed Narmashiri

Academic Editor

PLOS One
